# Long-Term Follow-Up of Custom-Made Porous Hydroxyapatite Cranioplasties: Analysis of Infections in Adult and Pediatric Patients

**DOI:** 10.3390/jcm13041133

**Published:** 2024-02-17

**Authors:** Francesca Carolina Mannella, Francesca Faedo, Marta Fumagalli, Giuseppe Danilo Norata, Ismail Zaed, Franco Servadei

**Affiliations:** 1 Department of Pharmacological and Biomolecular Sciences “Rodolfo Paoletti”, Università degli Studi di Milano, 20133 Milan, Italy; marta.fumagalli@unimi.it (M.F.); danilo.norata@unimi.it (G.D.N.); 2 Department of Biomedical Sciences, Humanitas University, 20072 Milan, Italy; franco.servadei@hunimed.eu; 3 Department of Neurosurgery, Neurocenter of the Southern Switzerland, Regional Hospital of Lugano, CH-2900 Lugano, Switzerland; ismail.zaed@eoc.ch; 4 Department of Neurosurgery, IRCCS Humanitas Research Hospital, 20089 Milan, Italy

**Keywords:** cranioplasty, porous hydroxyapatite, complication, infection

## Abstract

In neurosurgery, cranioplasty (CP) stands as a pivotal surgical intervention, particularly following head trauma or various neurosurgical interventions. This study scrutinizes the intricacies of CP, emphasizing its prevalence and associated complications, with a specific focus on custom-made porous hydroxyapatite (PHA) implants. The investigation spans 687 patients (with 80 patients of pediatric age, less than 14 years old) across 26 neurosurgical centers in five European countries. Methodologically, this study delves into patient characteristics, complications, and infection data through a comprehensive post-marketing on-site surveillance approach. Notably, infections emerged as the primary complication, affecting 41 patients (6% of implants) with a clear distinction in onset patterns between pediatric (with more infections, 10% versus 5.4% in adults and an earlier onset of complications) and adult populations. Out of these 41 cases, cranioplasty explantation was required in 30 patients, 4.4% of the total population. Furthermore, bifrontal decompression correlated with a significantly elevated infection risk as compared to unilateral decompression (12.5% versus 5.1%) which remains after the examination of possible confounding factors. These findings provide substantial insights into the complexities of CP, suggesting the necessity for tailored strategies in pediatric and adult cases and cautioning against bifrontal decompressions. Despite acknowledging limitations and calling for prospective studies with long term follow-up, this research advances our understanding of the use of PHA CP, guiding clinical decision-making and emphasizing the importance of customized approaches for diverse patient cohorts.

## 1. Introduction

In the field of neurosurgery, cranioplasty (CP) is a common surgical procedure performed worldwide. It is the last surgical step of a long healing pathway that many patients undergo after suffering from head trauma or other neurosurgical diseases, such as brain and bone tumors, infections, and vascular anomalies. It consists of the reconstruction of skull integrity by means of either an autologous bone flap or a heterologous implant in patients who present with a skull defect, mostly due to a previous decompressive craniectomy after traumatic brain injury.

CP is associated with a relatively high complication rate, the most common being post-operative bone flap or implant infection [1]. Many questions remain open on how to reduce these complications, such as timing, technique, and the material of the CP. Complications lead to increased length of hospital stay, increased costs, and often the need for a reintervention, ultimately leading to a less favorable outcome for the patient. The infection rate ranges from 1.4% to 24.4% [2,3,4], with established risk factors being poor pre-operative neurological status [5,6], trauma patients [7], bifrontal CP [7], diabetes [8,9], large skull defect size [9], and VP shunt [10].

A factor believed to affect the infection rate is the material used. Autologous bone is the most common material used and remains the gold standard for CP [11]. Nevertheless, autologous bone has been found to have a high infection rate in different series, and it also carries a risk of bone resorption [12]. In contrast with these findings, which are prevalent in the literature, a recent American National study showed that often heterologous material has a higher rate of complications as compared to autologous bone [13]. The other main materials used for CP are titanium, hydroxyapatite (HA), polymethacrylate (PMMA), and polyetheretherketone (PEEK). There are some new and less investigated materials used for CP, such as fiberglass and composites (titanium–HA composites and Cap-Ti) [14].

HA is a porous bioceramic that has biomimetic characteristics of bone, giving it its peculiar osteo-integrability feature [15,16]. Custom-made technology allows a higher rate of osteointegration and a good aesthetic outcome. Custom-made porous hydroxyapatite (PHA) CP has been shown in some previous studies to possibly have a lower infection rate with respect to other heterologous materials [17,18], but more evidence is needed to prove it.

In this study, the authors focused on custom-made PHA CP, collecting post-marketing data, and performing on-site interviews in 26 centers across five EU countries. The data collected included patient characteristics, the type and frequency of complications, and more detailed information on cases of infection and explants. This study represents the largest clinically based data collection on PHA CP (687 cranioplasties) and has a long follow-up. In order to compare our data with recent data concerning other materials, the authors performed a literature review of articles published between 2018 and 2022 reporting the infection rate after CP surgery with various materials.

## 2. Materials and Methods

The study includes 687 patients who underwent PHA cranioplasty (CustomBone Service, FinCeramica Faenza Spa, Faenza, Italy), whose clinical data were collected in the PMCF database from the manufacturing company. All cases were custom-made PHA since ceramics cannot be modelled on the surgical table.

The current Medical Devices Regulation (EU) 2017/745 (MDR) considers the clinical follow-up of medical devices on the market as a process aimed at continuously updating the clinical evaluation of a device. For this reason, proactive vigilance/surveillance activities are periodically carried out, with the aim of maintaining the long-term follow-up safety of the implanted devices. This activity, as stated in annex X 1.1-quarter of Directive 93/42/EEC (amended by Directive 2007/47/EC) and guidelines on medical devices (MEDDEV 2.7/1 rev.4; MEDDEV 2.12/1 rev.8), started in December 2018. To reach this goal, a clinical protocol accompanied by specific case report form (a copy of the protocol is available in Supplementary Materials Text File S1) was prepared and submitted to the CustomBone Service users for filling and collecting surveillance data. The protocol was designed as a retrospective, non-interventional study and the study population was composed of all the subjects as defined in CustomBone Service instruction for use. No specific inclusion or exclusion criteria were identified.

Surgeons’ informed consent for data elaboration and analysis was obtained for each device at the time of request/ordering to the manufacturer. Therefore, this surveillance activity did not require any ethic committee approval from the sites that contributed for data collection. Aggregate data were anonymized before the analysis.

Among all institutions that use custom-made PHA, 26 neurosurgical European centers were selected. The centers and neurosurgeons involved in the study are summarized in Table S1 in Supplementary Materials. Each center participating in the study was visited by a CRO collaborating with our institutions and the clinical protocols were filled with the aid of the neurosurgeons involved in the procedure as reported in previous studies.

Data were collected only from a review of medical records and the data collection was conducted in accordance with the 1964 Declaration of Helsinki and its later amendments or comparable ethical standards: Good Clinical Practices (GCP—ICH E6) defined in recent European directives and ICH E9 for statistical methods.

The data collection procedures were limited to the review of already existing medical records. For each patient, baseline characteristics (i.e., country, gender, age at the time of CP, initial pathology, reason for CP, skull defect localization, line of treatment, adverse events and complications, explants, and follow-up from the time of CP) were reviewed.

The data from the clinical follow-up up to the recorded complications were also registered and discussed with the surgeon in charge of the patient.

The aim of this study was to define the scenario in which infections develop after CP with custom-made PHA. To this purpose, clinical data of patients who underwent CP with CustomBone (PHA implants) were analyzed, also exploiting the FinCeramica database, using SPSS Statistics Version 28.0 (IBM Corp., Armonk, NY, USA, Released 2021). Statistical analyses were assessed using Chi-square test by employing Prism 9 software (GraphPad, San Diego, CA, USA), while binary logistic multivariate analysis was performed using SPSS Statistics (IBM Corp. Released 2021. IBM SPSS Statistics for Macintosh, Version 28.0. Armonk, NY, USA: IBM Corp.).

## 3. Results

### 3.1. PMCF Database Description

The PMCF database includes the clinical data of 687 patients who underwent cranioplasty (CP) utilizing the CustomBone Service device, as summarized in Table 1. The average follow-up period was 25.6 months (from 6 month to 60 months).

The database population was stratified into two groups: the pediatric group, comprising 80 patients (11.6%) aged between 2 and 13 years, and the adult group that included 607 patients (88.4%) aged 14 years and older. Specifically, 50 patients (62.5%) in the pediatric group were male, and 30 patients (37.5%) were female. In the adult group, 411 patients (67.7%) were male, and 196 patients (32.3%) were female.

Concerning the initial pathology for which CP was performed, trauma was the most common factor, accounting for 417 cases (60.7%), followed by vascular disorders (*n* = 118, 17.2%) and tumors (*n* = 118, 17.2%). A subgroup of 20 patients (2.9%) presented congenital malformations, while the remaining 14 patients (2%) underwent CP due to other pathologies.

The majority of patients underwent CP with CustomBone implants as the first-line treatment (*n* = 522, 76%), while 165 subjects (24%) received the device as a second-line treatment. Second-line treatment means patients who received a cranioplasty with another material which was explanted because of complications and subsequently were re-operated with PHA cranioplasty.

In terms of the localization of the cranial defect, 584 patients (85%) underwent CP in the fronto-parieto-temporal region, and 56 patients (8.2%) in the bifrontal region, while 47 subjects (6.2%) underwent CP in other regions.

Table 2 shows the complications reported in the PMCF database. Of the 687 patients analyzed, 80 (11.6%) reported complications. Specifically, infections were reported by 41 patients (6% of the total), fractures by 17 patients (2.5%), displacements by 7 patients (1%), and 15 patients (2.2%) reported other complications.

In more detail, we reported complications in pediatric and adult populations. Infections affected 8 children (10%) and 33 adults (5.4%), fractures were observed in 12 children (15%) and 5 adults (0.8%), and displacements occurred in 3 children (3.8%) and 4 adults (0.7%). Notably, other complications (*n* = 15, 2.5%) were exclusively reported in the adult population. Statistically significant differences between the two populations were in the rate of infection and the presence of post-implantation fractures, both more frequent in children.

### 3.2. Infections

Infections emerged as the most common complications reported in the PMCF database, constituting 41 cases (6% of total implants). All cases of infections, including cases necessitating implant removal post infection, were analyzed.

Here, the 41 patients were divided into two groups according to the infection onset: 26 patients (3.8%) developed infection within 2 months after CP (early infections), while in 15 cases (2.2%) infections were reported after 2 months from CP (late onset). Out of the 41 reported complications, implants were removed in 30 cases, representing 4.4% of the total number of devices implanted and 73% of the infected cases. In 11 cases, 10 of which were treated with long-course antibiotics, the cranioplasty was not removed despite the initial infection. Table 3 summarizes the data stratified for initial pathology, line of treatment, and localization of the CP.

**Figure 1:**
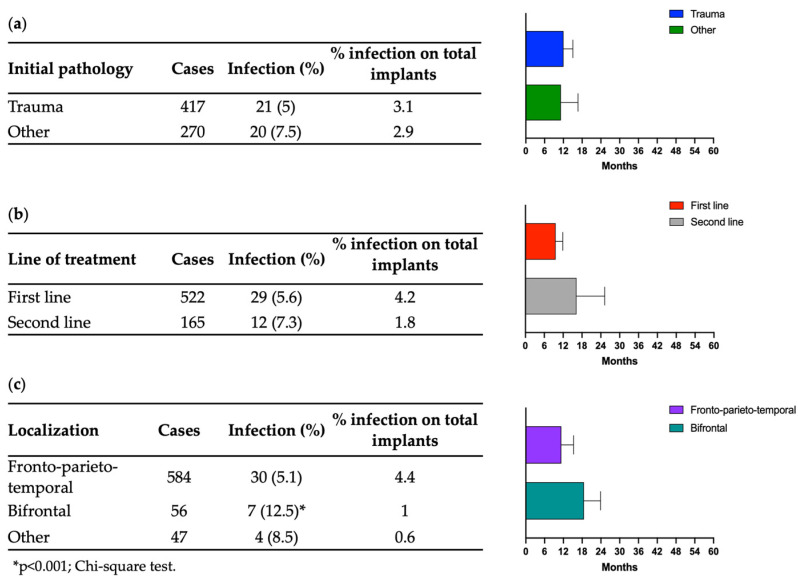
(**a**) Infection onset after trauma compared to other initial pathologies. (**b**) Comparison of the infection onset after first-line CP and second-line CP. (**c**) Comparison of the infection onset after CP in the fronto-parieto-temporal and bifrontal regions.

Furthermore, the onset of infections was evaluated considering the initial pathology, line of treatment, and localization of the cranial defect. Among 417 patients with trauma, infections were observed in 21 patients (5% of all trauma cases), with an average onset time of 12 months, compared to 11 months for patients with other initial pathologies (Figure 1a). Figure 1b delineates the onset of infection in patients who underwent CP as a first- or second-line treatment. Among the 522 patients in the first-line treatment category, 29 (5.6%) developed infections, with an average onset time of 16 months. In the second-line treatment group comprising 165 patients, 12 individuals (7.3%) experienced infections, with an average onset time of 10 months. Also, the explantation rate was similar between first- and second-line treatment both for early and late surgeries. Regarding cranial defect localization, we focused on the fronto-parieto-temporal and bifrontal regions. Of the 584 patients undergoing CP in the fronto-parieto-temporal region, 30 subjects (5.1%) developed infections, with an average onset time of 11 months. In contrast, among the 56 patients with bifrontal CP, 7 experienced infections, with an average onset time of 18.5 months (Figure 1c). The difference between the two different types of cranial decompression is statistically significant, with more infections in bifrontal craniectomies.

Moreover, we performed a binary logistic multivariate analysis to investigate whether age, initial pathology, and line of treatment differ in subjects who experienced infection following bilateral decompression vs. those who experienced an infection following cranioplasty in the fronto-parieto-temporal region. Neither age nor the initial pathology nor the line of treatment differed between the two groups, thus excluding a major role for these factors in determining the increased risk of infection observed in patients who had a bilateral decompression.

**Figure 2:**
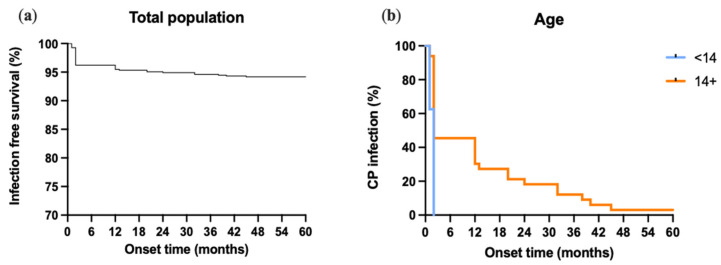
(**a**) Infection-free survival in total PMCF database population. (**b**) Infection onset after CP in adult (14+) and pediatric (<14) populations.

As mentioned above, this study identified 41 infections within the patient population. Further analyses were performed using the Kaplan–Meier method. Notably, 94% of all patients remained infection-free, with the remaining 6% experiencing an infection. Interestingly, 2/3 of patients who developed infections did so within the initial 6 months following CP (Figure 2a). In more detail, Figure 2b shows the infection rate after CP in the pediatric and adult populations. In the pediatric group, nearly 40% of infections happened within the first month post CP, while the remainder occurred after the second month. Contrastingly, among adult patients, 55% of infections emerged 2 months after CP, while the remaining 45% developed 12 months and later post CP. Comparing the group of pediatric patients with the group of adult patients, it is evident that children develop complications significantly earlier than adults.

## 4. Discussion

Decompressive craniectomy is widely used as a “second-tier” therapy to control refractory intracranial pressure. In patients surviving after severe brain damage, cranioplasty is a critical stage of cranial defect reconstruction. It has already been widely shown that cranial reconstruction is necessary not only for cosmetic reasons, but also to improve cerebral blood flow circulation and the overall neurological status [19].

Despite being used for centuries, cranial reconstruction has been extraordinarily advanced not only in terms of the technical procedures offered to the patients, but also in terms of the different heterologous materials that can be used. Such necessity has been raised by the significant rate of complications of the autologous bone, not only in terms of post-operative infection, but most importantly for the rate of bone reabsorption, which can be up to 50% in the pediatric population [20].

The most common heterologous materials used for cranioplasty are polymethacrylate (PMMA), titanium, polyetheretherketone (PEEK), and hydroxyapatite (PHA). If we focus on the complication rate in recent studies, it ranges around an average of 18% (PMMA), 13% (PEEK), 11% (titanium), and 10% (PHA), with no statistically significant difference among the materials. The conclusion of an Editorial published in 2017 that “the cranioplasty still need an ideal material and a surgical timing” is probably valid even nowadays [21].

The most important complication is post-operative infection, which is the cause of 50 to 70% of all cranioplasty explantation. Even if post-operative infections can be reduced with simple maneuvers like wound healing protocols [22] independent from the material used, in this paper we focused on the complication rate and in particular on the infection rate of a large series of European patients where a PHA cranioplasty was implanted.

The peculiarities of these casistics are the follow-up (median 25.6 months) and the on-site collection of data about complications, which is not common in multicentric studies where data are usually based on adverse events spontaneously reported to the material producers [16] or obtained through participation in national multicenter studies [23].

In order to compare our study with previously published series, we have included patients with different diagnosis such as cranial decompression for trauma, vascular reasons, or tumors. No correlation has been found in our series between the rate of infection and the original diagnosis responsible for the craniotomy. Riordan et al. published their retrospective experience where they found that sex, history of previous infection, history of craniectomy for trauma, and cranioplasty size were not statistically correlated with a higher rate of infection [24]. On the contrary, in an adult series, Giese et al. found that post-ischemic patients had a higher rate of infections. This is probably due to the higher number of cranial decompressions for ischemia in Germany as compared to other European countries where trauma is prevailing like in our casistics [25].

Surprisingly, no statistical difference has been found in the rate of complications in patients who received PHA cranioplasty as first- or second-line treatment, whereas it should be expected from previous reports in the literature [26], where a higher rate of post-operative complications in second-line patients mainly correlated with the generally poorer conditions of the patients.

In this paper, the authors aimed to better define the short- and long-term post-operative complications and in particular infections in PHA cranioplasty. According to this study, there was a 6% rate of infection: it was 10% in the pediatric population and 5.4% in the adult one, data which are statistically significant. The rate of infection for the material in adult patients is similar to the rate published in recent multicenter studies on PHA for adults [26] and children [27] and it is lower than other multicentric studies from France [28]. Out 41 infected cranioplasties, 30 (73% of all infections, 4.4% of all implanted devices) were explanted, which is also in line with previous reports [17]. If we compare these data with recently (from 2018 to 2022) published studies in adults on other heterologous materials, we see an infection rate from 2 to 23% for titanium (mean of all papers 7%) [29,30], from 0 to 42% for PMMA [29,31] (mean 15%), and from 3 to 43% for PEEK [32,33] (mean 9.2%). Using the same method, HA will have an infection rate from 0 to 21% [15,34], mean 8.3%. The difference in the published data about the materials is due to the longer follow-up for PHA patients, with 8 papers out of 11 with a follow-up longer than 1 year.

A very recently published paper [14] showed in an adult series a higher infection rate with PEEK and autologous bone cranioplasty as compared to PMMA and CaP-Ti (constituted by a net of titanium with PHA). There were only two cases of PHA and therefore this material in isolation could not be included in the analysis. 

A systematic review in pediatric-age patients includes a total of 20 case series resulting in 544 patients. Out of the total number of patients, 422 (77.6%) received heterologous materials and 122 (22.4%) received autologous bone. A rate of infection of 10% was reported for polyetheretherketone (PEEK), as compared to 4% for PHA and 2% for titanium cranioplasty but with fewer patients [35].

As also already reported in this review, the lengths of follow-up are different, with longer follow-ups in PEEK and PHA patients. 

It has been noted that we showed in the same casistics a different timing for the occurrence of infections between children and adults for the first time: in children, the vast majority of infection were identified in the first months, whereas in adults the infections were spread over a period of months if not years. This has a practical clinical side: the follow up in adults should never be limited to the first months after cranioplasty insertion. 

We also found that 26 patients (3.8%) developed infection within 2 months (early infections), while in the remaining 15 cases (2.2%), infections were reported after 2 months (late onset). Out of the 41 reported complications, implants were removed in 30 cases, data which are statistically significant, representing 4.4% of the total number of devices implanted. A lower rate of explantation in the late-onset group could be explained by the fact that PHA cranioplasty, as well as CaP-Ti cranioplasty which contains HA [14], is known to promote angiogenesis and osteointegration with the surrounding skull. In this context, a prolonged antibiotic therapy also could be effective in reducing the bacterial activity at the local level without device-explantation-related costs and hospital stays [36], as happened in 11 of the 41 cases of infection.

Concerning the presence of skull fractures, the global incidence is 2.5% with 0.8% in adults and 15% in children. PHA is fragile when inserted and requires osteointegration (over 6 months at least) to become stronger and resistant to bumps. When inserted in children or in non-collaborating adults, the risk of fractures significantly increases.

Another factor affecting infection occurrence was the type of decompression: bifrontal decompression was more prone to infections (12.5%) than unilateral fronto-temporo-parietal decompression (5.1%). There are many papers published on autologous bone, PHA, titanium, and PMMA confirming our findings [16,37,38,39]. These infections are possibly due to a longer skin incision, to a lower availability of temporal muscle for coverage, to a longer perioperative time [37], and as a main factor in our experience to the opening of the frontal sinuses without a proper closure during emergency surgery. Bifrontal decompressive craniectomy was indicated in all cases of secondary decompression in refractory ICP in diffuse injured patients in a recent randomized trial [40] and in 63% of the cases of refractory ICP in focal and diffuse lesions patients [41]. Our casistics show a much lower number of bifrontal decompressions (57 cases/687 patients, 8.3%) probably due to the overwhelming number of primary decompressions (with hematoma evacuation). In these cases, a recent randomized study of decompression in acute subdural hematoma showed the almost sole use of a unilateral bone flap for cranial decompression [42].

### 4.1. Practical Recommandations

- PHA cranioplasty needs osteointegration to become strong enough to avoid cranial fractures in the case of a second trauma. Implantation in children and non-collaborating adults should be accompanied with patients keeping protective helmets on for at least for six months.
- In selected cases of post-operative infections, a course of medical therapy can avoid the explantation of the prothesis.
- As for other heterologous materials, bifrontal cranial reconstruction carries an increased risk of post-operative infections and should be avoided whenever possible.

### 4.2. Study Limitations

Despite the best efforts, the present study is characterized by some limitations. Of note, despite the large case series and multicenter design, the present study had no control arm to compare the results of PHA CP with autografts or other synthetic CP. 

In addition, this study was not designed to specifically compare outcomes between early and late CP and between children and adults. Further prospective studies are advisable in the future.

Moreover, this study was only conducted in European Centers, since out of more than 9000 PHA implants, 98% were implanted, up to now, in European Countries. This will obviously limit the applicability of our findings to other continents where CPA cranioplasty is very often not yet available.

## 5. Conclusions

Our study on a single material for cranial reconstruction with s large series of European patients and a long follow-up shows that PHA is a feasible method of cranial reconstruction with some advantages related to possible osteointegration. Pediatric and adult patients should be examined (and published) in different casistics since the rate of complication (infections and fractures) and the timing of infections are totally different. Bifrontal cranial decompression should be avoided whenever possible since this technique carries a higher risk of infections over time.

Future research should prioritize prospective studies and long-term follow-up to provide more information about our observed complication rate and infection patterns.

## Figures and Tables

**Table 1:** Characteristics of patients included in PMCF database.

	Number	%
**Number of patients**	687	100
**Gender**		
Male	461	67.1
Female	226	32.9
**Age**		
Mean age	37.2	
Pediatric (2–13 years)	80	11.6
Male	50	62.5
Female	30	37.5
Adult (14+ years)	607	88.4
Male	411	67.7
Female	196	32.3
**Initial pathology**		
Trauma	417	60.7
Vascular disease	118	17.2
Tumor	118	17.2
Malformation	20	2.9
Other	14	2
**Line of treatment**		
First line	522	76
Second line	165	24
**Localization**		
Fronto-parieto-temporal	584	85
Bifrontal	56	8.2
Other	47	6.8

**Table 2:** Description of complications reported in PMCF database following cranioplasty.

Complications (*n* = 80)	Number	%
Global infection	41	6
Pediatric (2–13 years)	8 *	10
Adult (14+ years)	33 *	5.4
Global fracture	17	2.5
Pediatric (2–13 years)	12 *	15
Adult (14+ years)	5 *	0.8
Global displacement	7	1
Pediatric (2–13 years)	3	3.8
Adult (14+ years)	4	0.7
Other	15	2.2
Pediatric (2–13 years)	-	-
Adult (14+ years)	15	2.5

* *p* < 0.001; Chi-square test.

**Table 3:** Description of infection onset and explants following cranioplasty.

	Early Infections (*n* = 26; 3.8%)		Late Infections (*n* = 15; 2.2%)	
	Cases (%)	Explant (%)	Cases (%)	Explant (%)
**Initial pathology**				
Trauma	12 (1.8)	8 (1.2)	9 (1.3)	7 (1)
Vascular disease	9 (1.3)	7 (1)	2 (0.3)	1 (0.2)
Tumor	3 (0.4)	3 (0.4)	2 (0.3)	1 (0.2)
Malformation	2 (0.3)	2 (0.3)	-	-
Other	-	-	2 (0.3)	1 (0.2)
**Line of treatment**				
First-line	18 (2.6)	13 (1.9)	11 (1.6)	7 (1)
Second-line	8 (1.2)	7 (1)	4 (0.6)	3 (0.4)
**Localization**				
Fronto-parieto-temporal	20 (2.9)	15 (2.2)	10 (1.5)	8 (1.2)
Bifrontal	2 (0.3)	1 (0.2)	5 (0.7)	2 (0.3)
Other	4 (0.6)	4 (0.6)	-	-

## Data Availability

The original contributions presented in the study are included in the article and Supplementary Materials, and further inquiries can be directed to the corresponding authors.

## Supplementary Materials

The following supporting information can be downloaded at https://www.mdpi.com/article/10.3390/jcm13041133/s1: Text File S1: Surveillance protocol; Table S1: Centers and neurosurgeons involved in the study.
