# Supplementary material for: Long-Term Follow-Up of Custom-Made Porous Hydroxyapatite Cranioplasties: Analysis of Infections in Adult and Pediatric Patients
Source: J Clin Med. 2024 Feb 17;13(4):1133. doi: 10.3390/jcm13041133 (PMC10888657; doi:10.3390/jcm13041133)
Supplement: Supplementary file 1 [file jcm-13-01133-s001.zip › jcm-2854918-supplementary.pdf]

Table S1. European neurosurgical centres.

Country	City	Hospital	Neurosurgeons involved
Belgium	Ghent	Ghent University Hospital	G. Hallaert
France	Lyon	Hospices Civil de Lyon	C. Mottolese
	Paris	Hôpital Lariboisière	S. Chibbaro
	Strasburg	Hôpital de Hautepierre	S. Chibbaro
	Tours	Hôpital Bretonneau	P. Francois
Ireland	Dublin	Beaumont Hospital	D. Crimmins
		Temple Street children's hospital	D. Crimmins
Italy	Alessandria	Ospedale SS. Antonio e Biagio e Cesare Arrigo	A. Barbanera
	Ancona	Ospedali Riuniti	A. Di Rienzo, M. Luzi
	Aosta	Ospedale Regionale della Valle D'Aosta	A. Barbanera
	Bologna	Ospedale Bellaria	E. Serchi
		Ospedale Maggiore	E. Serchi
	Brescia	Spedali Riuniti	R. Stefini, C. Cereda
	Catania	Ospedale Cannizzaro	M. Fricia
	Gemona del Friuli	Ospedale San Michele	P.C. Parodi
	Genova	Ospedale Gaslini	M. Pavanello
	Legnano	ASST Ovest Milanese	R. Stefini
	Mantova	Ospedale Poma	B. Zanotti
	Napoli	Ospedale Santobono	P. Spennato, C. Ruggiero
	Padova	Ospedale Civile	R. Faggin,
	Palermo	Policlinico Universitario	F. Graziano
	Parma	Ospedale Maggiore	C. Iaccarino
	Perugia	Ospedale Santa Maria della Misericordia	G. Ghetti
	Roma	Bambino Gesù Children's Hospital	P. Palma
	Rozzano	Humanitas Research Hospital	F. Servadei
	Udine	Ospedale Santa Maria della Misericordia	B. Zanotti
	Vicenza	Ospedale San Bortolo	G.P. Zambon
Switzerland	Lausanne	Centre Hospitalier Universitaire Vaudois	R. Maduri

Document type: TDOC	Section: /	Number: 0522
Title: **Pro-active Post-Marketing Data Collection for CustomBone Service Implants**		Edition / Revision: 01/0

REVISION N°	*DATE*	*REVISION DESCRIPTION*
0	16/11/18	FIRST EDITION OF THE DOCUMENT (RIF. PDM/NE 091/18)
1		
2		
3		
4		
5		
6		
7		
8		
9		

ATTACHMENTS' LIST:

ATTACHMENT N°	*TITLE*
01	INVESTIGATOR AGREEMENT
02	POST- MARKETING DATA FORM
03	INTRA-OP ADVERSE EVENT FORM
04	POST-OP ADVERSE EVENT FORM

DOCUMENT DISTRIBUTION:

CONTROLLED COPY NUMBER:	*RECIPIENT:*	*SIGN/DATE*

DOCUMENT SIGNATURES

WRITTEN BY: CLINICAL RESEARCHER ASS. NEURO (VALENTINA CANELLA)	SIGNATURE:	DATE:
REVIEWED BY: MEDICAL MKTG MANAGER (ANGELO NATALONI)	SIGNATURE:	DATE:
REVIEWED BY: REGULATORY AFFAIRS ASSISTANT (VALENTINA BALBI)	SIGNATURE:	
REVIEWED BY: V. P. STRATEGIC DEVELOPMENT (CLAUDIO DE LUCA)	SIGNATURE:	DATE:
APPROVED BY: QUALITY ASSURANCE MANAGER (ROBERTA MARTINETTI)	SIGNATURE:	DATE:

SIGNATURES ON THE ORIGINAL COPY

	**DATE**	**TO BE REVISED WITHIN**	**SIGN**
**ENTRY INTO FORCE**	16/11/2018	/	
**REVISION**			
**REVISION**			

Document substituted on (date)	Sign/Date

**REFERENCE:**		
Filename:TDOC_0522_01-0_pro-active_PMS_CBS.docx	Archive: QA	Pag./Tot. Pag.: 1/15

TABLE OF CONTENTS

1 STUDY SUMMARY 3

2 LIST OF ABBREVIATIONS 4

3 INTRODUCTION 4

4 PROTOCOL RATIONALE 4

5 STUDY OBJECTIVES 5

5.1 Primary Objectives 5

5.2 Secondary Objectives 5

6 STUDY DESIGN AND METHODS 5

6.1 General Study Design 5

6.2 Total Number of Subjects 5

6.3 Study Population 5

6.3.1 Inclusion/Exclusion Criteria 5

7 STUDY PROCEDURES 5

7.1 Data Sources 5

7.2 Variable Abstraction 5

8 STATISTICAL PLAN 6

9 STUDY ADMINISTRATION 6

9.1 Data Collection and Management 6

9.2 Confidentiality 6

10 ETHICS AND REGULATORY CONSIDERATIONS 6

10.1 Compliance Statement 6

10.2 Compliance with GCPs 6

10.3 Patient Information 6

10.4 Patient Confidentiality 7

11 BIBLIOGRAPHY 7

12 ATTACHMENTS 7

01. INVESTIGATOR AGREEMENT 7

02. POST-MARKETING DATA FORM 7

03. INTRA-OP ADVERSE EVENT FORM 7

04. POST-OP ADVERSE EVENT FORM 7

INVESTIGATOR AGREEMENT 8

1 STUDY SUMMARY

**Title**	Pro-active Post-Marketing Data Collection for CustomBone Service (CBS) Implants
**Study Type**	Post-Marketing Surveillance Activity
**Study Design**	Retrospective, non-interventional, pro-active post-marketing surveillance activity
**Product**	CustomBone Service, a patient-specific medical device for cranioplasty
**Indication**	Reconstruction of cranial defects
**Study Duration**	Not Applicable
**Site Number(s)**	Not Applicable
**Objectives**	Primary objective of the present pro-active post-marketing surveillance activity is:<br>- the evaluation of the safety outcome, in terms of explantation rates (i.e. failure), of the hydroxyapatite (HA) implant CBS<br>- the verification of whether the causes of explantation are different from those commonly reported by the Manufacturer's Risk Analysis.<br><br>Secondary objectives include:<br>- the identification and evaluation of the residual risks associated with the medium/long-term use of the HA-based implant CustomBone Service.
**Number of Subjects**	Not Applicable
**Inclusion/Exclusion Criteria**	Not Applicable
**Parameters recorded from the patients' medical records**	As retrospective data collection, no interventions will be performed on patients. Data will be collected only as review of medical records.<br>For each CBS, patient-related parameters and device-related parameters will be analysed. Examples of parameters are reported below:<br>- patient's age<br>- gender<br>- date of craniectomy<br>- date of surgery with CBS<br>- indication for craniectomy/cranioplasty<br>- skull defect localization<br>- interval time between craniectomy and cranioplasty<br>- adverse event(s)<br>- follow-up (last visit) from surgery
**Surgical Interventions**	As retrospective data collection, no interventions will be performed on patients. Data will be collected only as review of medical records.

2 LIST OF ABBREVIATIONS

AE (adverse event): any untoward medical occurrence, unintended disease or injury or any untoward clinical signs (including an abnormal laboratory finding) in subjects, users or other persons whether or not related to the investigational medical device (ref. ISO 14155)

CBS: CustomBone Service

CRF: Case Report Form

FCF: Fin-Ceramica Faenza S.p.A.

HA: Hydroxyapatite

MD: Medical Device

PMS (Post-Marketing Surveillance): the practice of monitoring the safety of a medical device after it has been released on the market (see European Guidelines MEDDEV 2.12-1 Rev.8; SOP 03, Section 8.5)

SAE (Serious Adverse Event): Adverse event that:

a) Led to a death

b) Led to a serious deterioration in health that either:

1) Resulted in a life-threatening illness or injury, or

2) Resulted in a permanent impairment of a body structure or a body function, or

3) Required in-patient hospitalization or prolongation of existing hospitalization, or

4) Resulted in medical or surgical intervention to prevent life threatening illness or injury or permanent impairment to a body structure or a body function,

c) Led to fetal distress, fetal death or a congenital abnormality or birth defect (ref. ISO 14155).

3 INTRODUCTION

The CustomBone Service (CBS) is a patient-specific, implantable medical device made of Hydroxyapatite (HA). CBS is suitable for repair of cranial defects and is characterized by high porosity, chemical composition and structure resembling the mineral component of human bones. CBS implants are bio-mimetic and well tolerated by the patients. The device is designed and produced according to surgeon's specifications and based on the patient's 3D CT scan data. The implant shape is customized for every single patient following the dimension and features of his specific cranial defect.
The device has been implanted already in more than 5.380 patients (Report N. 0556) and it is supported by a large number of peer-reviewed clinical and pre-clinical publications [1–7]. In addition, pro-active vigilance/surveillance activities are periodically carried out as required by Regulatory Guidelines on medical devices (for more details, please refer to MEDDEV 2.7/1 rev.4), with the aim to keep verified the long-term follow-up safety of the devices in the implanted population.
For these purposes, this document was designed and will be submitted to the CBS users for filling and collecting post-marketing data.

4 PROTOCOL RATIONALE

The present protocol has been conceived as a pro-active, post-marketing surveillance activity, aimed at evaluating the safety outcomes of the bioceramic implant CBS and to identify eventual residual risks associated with the medium/long-term use of the HA-based implant.
Surgeons informed consent for data elaboration and analysis has been obtained for each device at the time of request/ordering to the Manufacturer. Therefore, for the reasons stated above, the present surveillance activity does not require any Ethic Committees approval from the Sites that will contribute for data collection.

Data will be collected only as review of medical records. The PMS data collection will be conducted in accordance with 1964 Helsinki declaration and its later amendments or comparable ethical standards.

5 STUDY OBJECTIVES

5.1 Primary Objectives

The purpose of this pro-active post-marketing surveillance activity is to determine the safety outcomes, in terms of explantation rates (i.e. failure), of the bioceramic implant CBS, and to verify whether the causes of explantation are different from those commonly reported by the Manufacturer's Risk Analysis.

5.2 Secondary Objectives

Secondary objectives include the identification and evaluation of the residual risks associated with the medium/long-term use of the HA-based implant CustomBone Service.

6 STUDY DESIGN AND METHODS

6.1 General Study Design

Retrospective, non-interventional, pro-active post-marketing surveillance activity.

6.2 Total Number of Subjects

N/A

6.3 Study Population

The study population is composed of all the subjects as defined in CBS Instruction for use.

6.3.1 Inclusion/Exclusion Criteria

No specific inclusion or exclusion criteria have been identified. The main aim of this pro-active activity is to collect all the data available for patients implanted with CBS, in order to improve the information spontaneously coming back from the market.

7 STUDY PROCEDURES

For this protocol, the procedures of data collection are limited to the review of already existing medical records. For each patient, baseline characteristics (i.e. gender, age at the time of cranioplasty procedure, indication for craniectomy/cranioplasty, skull defect localization, interval time between craniectomy and cranioplasty, adverse events and complications, follow-up from the time of cranioplasty, outcomes) will be reviewed.

7.1 Data Sources

Patients' medical records, including electronic medical records, will be used to collected data for the present post-marketing activity.

7.2 Variable Abstraction

Data sources are represented by medical records, including electronic medical records, which will be identified by the specific serial number associated to each medical device manufactured.
Example of data which can be abstracted from medical records are as follows (but not limited to):

- Gender,
- Age at the time of cranioplasty procedure,
- Indication for craniectomy/cranioplasty,
- Skull defect localization,
- Interval time between craniectomy and cranioplasty,
- Adverse events and complications,
- Follow-up from the time of cranioplasty, outcomes,
- Other parameters (where available).

8 STATISTICAL PLAN

All the analyses will be conducted on the whole collected data, if not otherwise specified. Only descriptive analyses will be provided.

9 STUDY ADMINISTRATION

9.1 Data Collection and Management

1. The Medical Device List identification will be provided by each site. The MD code number specific for each CustomBone Service will be used for a case-by-case identification. The required information on the safety reported on the CRF will be filled by the surgeon himself.
2. The CRF will be signed and dated for confirmation wherever required in the paper form. In case of loss of signature and date from the surgeons, the collected information will not be considered as evaluable and will not be recorded in the database file.
3. The filled CRF will be collected by Fin-Ceramica Clinical Department personnel. The selected clinical staff will be properly trained before the beginning of the present activity.

From the CRF to the excel file:

1. The database access will be allowed to the selected Fin-Ceramica personnel, exclusively intended for this data collection, for data recording. For the predisposed excel file, Fin-Ceramica responsible personnel will use a password-protected file for data entry.
2. From the excel file to the statistician: before sending the database to the statistician, the excel file will be "frozen" in order to avoid any data changes or loss-of-data.

9.2 Confidentiality

All data and records generated during this activity will be kept confidential in accordance with Institutional policies on subject privacy. The Investigator and other site personnel will not use such data and records for any purpose other than conducting the study. Safeguards are described under Data Collection and Management. The data will be saved to the Fin-Ceramica Faenza Head quarter.

10 ETHICS AND REGULATORY CONSIDERATIONS

10.1 Compliance Statement

The study complies with the Post-Marketing Surveillance obligations, according to the Medical Devices Directive 93/42/EEC amended with Directive 2007/47/EC. Collection, recording, and reporting of data will be accurate and will ensure the privacy, health, and welfare of subjects.

10.2 Compliance with GCPs

The protocol will be conducted in compliance with the Declaration of Helsinki, Good Clinical Practices (GCP – ICH E6) defined in ISO 14155 (current edition) and ICH E9 for statistical methods.
It is worth noting that the present protocol is designed *as* a "Retrospective, Post-Marketing Surveillance Study". Patient and surgeon informed consent for data elaboration and analysis has been obtained for each device at the time of request/ordering. Therefore, for the reasons above, the present surveillance activity does not require any Ethic Committees approval from the Sites that will participate spontaneously in the activity.

10.3 Patient Information

Patient and surgeon informed consent for data elaboration and analysis has been obtained for each device at the time of request/ordering. If needed, it is the Surgeon's responsibility to collect additional patient informed consent for the treatment of confidential data.

10.4 Patient Confidentiality

All information and data concerning patients or their participation in this study will be anonymous and considered confidential. All data used in the analysis and reporting of this study will be used in a manner without identifiable reference to the enrolled patients.
Each physician shall keep in his/her institution a list of confidential correspondence between the patients' names and their study identification codes.
The right of access of patients' shall be exercised exclusively through the interventional neurosurgeons, since they will be the only persons able to establish correspondence between the patient's name and the study identification codes.

11 BIBLIOGRAPHY

1. Fricia, M. *et al.* Osteointegration in Custom-made Porous Hydroxyapatite Cranial Implants: From Reconstructive Surgery to Regenerative Medicine. *World Neurosurg.* **84,** 591.e11-16 (2015).
2. Hardy, H. *et al.* [Clinical and ossification outcome of custom-made hydroxyapatite prothese for large skull defect]. *Neurochirurgie.* **58,** 25–29 (2012).
3. Iaccarino, C. *et al.* Preliminary Results of a Prospective Study on Methods of Cranial Reconstruction. *J. Oral Maxillofac. Surg. Off. J. Am. Assoc. Oral Maxillofac. Surg.* **73,** 2375–2378 (2015).
4. Lindner, D. *et al.* Cranioplasty using custom-made hydroxyapatite versus titanium: a randomized clinical trial. *J. Neurosurg.* 1–9 (2016). doi:10.3171/2015.10.JNS151245
5. Staffa, G. *et al.* Custom made bioceramic implants in complex and large cranial reconstruction: a two-year follow-up. *J. Cranio-Maxillo-fac. Surg. Off. Publ. Eur. Assoc. Cranio-Maxillo-fac. Surg.* **40,** e65-70 (2012).
6. Stefini, R. *et al.* Use of 'custom made' porous hydroxyapatite implants for cranioplasty: postoperative analysis of complications in 1549 patients. *Surg. Neurol. Int.* **4,** 12 (2013).
7. Stefini, R. *et al.* The efficacy of custom-made porous hydroxyapatite prostheses for cranioplasty: evaluation of postmarketing data on 2697 patients. *J. Appl. Biomater. Funct. Mater.* **13,** e136-144 (2015).

12 ATTACHMENTS

01. INVESTIGATOR AGREEMENT
02. POST-MARKETING DATA FORM
03. INTRA-OP ADVERSE EVENT FORM
04. POST-OP ADVERSE EVENT FORM

INVESTIGATOR AGREEMENT

Title: Pro-active Post-Marketing Data Collection for CustomBone Service implants

Short title: Retrospective Review of Existing Data

Prepared by: Fin-Ceramica Faenza S.p.a.

INVESTIGATOR AGREEMENT

I hereby confirm that I have read and understood the protocol, the study objectives, and the case report form. I agree to provide post-market surveillance data for this retrospective activity, as according to the present protocol and in compliance with:

- the ethical principles stated in accordance with the ICH Guidelines;
- the applicable guidelines for Good Clinical Practices;
- the applicable laws and regulations for the country of the study site for which I am responsible;

Signed by:

Name and surname: ________________________________

Date: ____

Signature: ____

CONTACT INFORMATION

Study Site: _______________________________________

Patient: (three letters Name, three letter surname) _ _ _ / _ _ _	CustomBoneService Code: ______________________________	Principal prosthesis ☐<br>Back-up prosthesis ☐

Doctor (name/surname) ________________________________

E-mail: ________________________________

Hospital/Clinic: ________________________________

Address Hospital ________________________________

Country: ________________________________

Data Protection Information	
The Doctor declares and certifies that the patient or other individual legitimately authorized to represent the patient, has been given the specific consent concerning the gathering and handling of personal and sensitive data indicated in this request and the accompanying medical documentation needed for designing, producing, supplying and post-supply surveillance of the custom-made device in porous hydroxyapatite.	☐
The Doctor declares to be compliant with the provisions of the current legislation regarding the protection of patient's personal data	☐
Doctor signature: ____________________________	Date: ____________________________________

PATIENT DEMOGRAPHIC DATA	
Gender	|__| Male |__| Female
Age	|__ __|
Aetiology	☐ Trauma<br>☐ Vascular<br>☐ Tumour<br>☐ Malformation<br>☐ Other
Reason for Device Implantation	☐ Decompression craniectomy<br>☐ Comminuted fracture<br>☐ Tumor resection<br>☐ Autologous bone reabsorption/infection<br>☐ Other material rejection<br>☐ Malformation<br>☐ Other
Localization of Skull Defect	☐ Fronto-parieto-temporal<br>☐ Frontal<br>☐ Fronto-temporal<br>☐ Fronto-parietal<br>☐ Parietal<br>☐ Cranial vault<br>☐ Bifrontal<br>☐ Temporal<br>☐ Occipital<br>☐ Parieto-temporal<br>☐ Other
Side	☐ Left<br>☐ Right<br>☐ Other
Craniectomy Surgery Date	__ __ /__ __ /__ __ __ __ (dd/mm/yyyy)
Cranioplasty Reconstruction Date	__ __ /__ __ /__ __ __ __ (dd/mm/yyyy)
Cranioplasty was performed by Dr.	____________________________
Any intra/post-op AE occurred?	|__| No<br>|__| Yes *(if yes please fill in: Attachment 3: Intra-Op AEs; Attachment 4: Post-Op AEs)*

FOLLOW-UP VISITS	
**FU 30 days**	|__| Yes |__| No
**FU 6 months (1-9 m)**	|__| Yes |__| No
**FU 12 months (9-15 m)**	|__| Yes |__| No
**FU 24 months (15-24 m)**	|__| Yes |__| No
**FU 3 years (+/- 3 m)**	|__| Yes |__| No
**FU 4 years (+/- 3 m)**	|__| Yes |__| No
**FU > 4 years (+/- 3 m)**	|__| Yes |__| No

Patient: (three letters Name, three letter surname) _ _ _ / _ _ _	CustomBone Service Code: ______________________________	Principal prosthesis ☐<br>Back-up prosthesis ☐

INTRA-OP ADVERSE EVENT		
**Description**		
☐ Implant Device Fracture		
☐ Device does not fit the cranial defect (i.e. because of wrong dimensions/shape of the device)	Specify: _________________________________	
☐ Project error (i.e. mirroring)	Specify: _________________________________	
☐ Surgical error	Specify: _________________________________	
☐ Other surgery-related complications	Specify: _________________________________	
☐ Shipping error	Specify: _________________________________	
☐ Other AE	Specify: ___________________________________	
**Additional Notes**		
Adverse Event Treatment	**Treatment**	
	Yes<br>(please specify how)	☐ Substitution with back-up<br>☐ Surgery resolved with addition of a bone graft<br>☐ Other material (*specify*_______________)<br>☐ Other solution (*specify*_______________)
	No	☐
	Not yet<br>(please specify why)	☐ Monitoring (i.e. *the device was left in place*)<br>☐ Waiting for a new CBS device<br>☐ No Back Up available<br>☐ The patient's health got worsened (i.e. *swelling*)
AE-Outcome	☐ Resolved<br>☐ Unknown<br>☐ Failure	
Incident	☐ Yes ☐ No	
Relationship with the Device	☐ Related<br>☐ Possibly related<br>☐ Not Related<br>☐ N/A	

Relationship with the Surgery	☐ Related<br>☐ Possibly related<br>☐ Not Related<br>☐ N/A
Is the clinical case part of a Clinical Study?	☐ Yes ☐ No<br>If yes, please provide the following information:<br>|__| Sponsor-Initiated Clinical Study<br>|__| Investigator-Initiated Clinical Study
Has the clinical case been presented to congresses/poster sessions/etc..	☐ Yes ☐ No<br>If yes, please provide information:<br>Congress year ___________________________<br>Congress Event Name _____________________

Further details and comments:

____

____

Date of completion: |__|__| |__|__| |__|__|__|__| Signature: ____________________________
dd mm yyyy

Patient: (three letters Name, three letter surname) _ _ _ / _ _ _	CustomBoneService Code: ________________________________	Principal prosthesis ☐<br>Back-up prosthesis ☐

**POST-OP ADVERSE EVENTS**					
Did any AE occur?	|__| Yes |__| No				
Adverse Event onset	__ __ /__ __ /__ __ __ __ (dd/mm/yyyy)				
**AE Description**					
☐ Fracture	☐ Spontaneous	☐ After trauma			
	☐ Simple fracture	☐ Comminute			
	☐ Displaced fracture	☐ Other, please specify:________________			
	☐ ≤ 6 months<br>☐ >6 months/ < 1 year<br>☐ ≥ 1 year				
☐ Infection	**Surgical Site Infection**<br>☐ Superficial (skin)<br>☐ Deep: ☐ Meningeal ☐ Medical device ☐ Other<br>☐ Local: ☐ Loco-regional ☐ Systemic				
	**Microbiological exam**				
	☐ bacterial	☐fungus	☐other	☐Unknown	☐ not done
	☐ ≤ 6 months<br>☐ >6 months/ < 1 year<br>☐ ≥ 1 year				
☐ Mobilization	☐ Device Lowering ☐ Device raising ☐ Other: ____________				
☐ Tumour recurrence					
☐ Subdural Hematoma					
☐ Epidural Hematoma					
☐ Wound retraction					
☐ Slow Wound Healing					
☐ Epileptic Seizure					
☐ Other AE	Specify: ____				

**Seriousness**		
☐ SAE - Serious Adverse Event<br>*(any untoward medical occurrence that results in death/is life-threatening/requires inpatient hospitalization or causes prolongation of existing hospitalization/results in persistent or significant disability/incapacity/is a congenital anomaly/birth defect/requires intervention to prevent permanent impairment or damage*		
☐ AE - Adverse Event		
**Severity**		
Grade 1	Asymptomatic or mild symptoms; clinical or diagnostic observations only; no intervention indicated	☐
Grade 2	Moderate; minimal, local or non-invasive intervention	☐
Grade 3	Severe: Symptom(s) causing severe discomfort and significant impact of the patient's usual activity and requires treatment; hospitalization or prolongation of hospitalization indicated; disabling	☐
Grade 4	Life threatening: urgent intervention indicated.	☐
Grade 5	Death related to an AE	☐

**Additional Notes**		
**Was the Device explanted?**		
☐ Yes	☐ No	
AE-Treatment	Yes<br>(please specify how)	☐ Reoperation with back-up CustomBone prosthesis<br>☐ Reoperation with new CustomBone prosthesis<br>☐ Reoperation with the same CustomBone prosthesis (repositioning)<br>☐ Treated in situ with antibiotics (in case of infection)<br>☐ Reoperation with other materials/cranioplasty devices<br>☐ Hospitalized or prolonged hospitalization<br>☐ No other reoperation needed<br>☐ Monitoring<br>☐ Unchanged
	No	☐
	Not yet (please specify why)	☐ Patient monitoring ("wait and see")<br>☐ no other reoperation needed
AE Outcome	☐ Resolved<br>☐ Unknown<br>☐ Failure	

Event classified as Incident	☐ Yes ☐ No
Relationship vs Device	☐ Related<br>☐ Possibly related<br>☐ Not Related<br>☐ Not clear
Relationship with the procedure / surgery	☐ Related<br>☐ Possibly related<br>☐ Not Related<br>☐ Not clear
Is the clinical case part of a Clinical Study?	☐ Yes ☐ No<br>If yes, please provide the following information: |__| Sponsor-Initiated Clinical Study |__| Investigator-Initiated Clinical Study
Has the clinical case been presented to congresses/poster sessions/etc..	☐ Yes ☐ No<br>If yes, please provide information:<br>Congress year ___________________________<br>Congress Event Name _____________________
Additional Notes	— — — — — — — — —

**Adverse Event Status**			
☐ Closed/Resolved		Start date	|__|__| |__|__| |__|__|__|__| dd mm yyyy
		Stop date	|__|__| |__|__| |__|__|__|__| dd mm yyyy
☐	Closed (Stop date unknown)		
☐	Still ongoing/in progress		

Further details and comments:

____

____

Date of completion: |__|__| |__|__| |__|__|__|__| Signature: ____________________________
dd mm yyyy
